# Functional Movement Patterns, Spinal Posture and Prevalence of Musculoskeletal Symptoms among Elite Ice Hockey Players: A Cross Sectional Study

**DOI:** 10.5114/jhk/161548

**Published:** 2023-04-20

**Authors:** Małgorzata Grabara, Anna Bieniec

**Affiliations:** 1 Institute of Sport Science, Jerzy Kukuczka Academy of Physical Education in Katowice, Katowice, Poland.; 2 Department of Health-Related Physical Activity and Tourism, Jerzy Kukuczka Academy of Physical Education in Katowice, Katowice, Poland.

**Keywords:** training programs, FMS, athletes, asymmetries, sagittal spinal curvatures

## Abstract

The aim of this study was to evaluate functional movement patterns and spinal posture of elite ice hockey players and to examine the association between spinal posture, prevalence of musculoskeletal symptoms and Functional Movement Screen (FMS^TM^) scores. The study included 86 elite male ice hockey players aged 18 to 38 years. Sagittal spinal curvatures were measured with a Saunders digital inclinometer, and functional movement patterns were assessed by the FMS^TM^. Spinal posture of the studied ice hockey players was characterized by normal kyphosis (46%) or hyperkyphosis (41%) and decreased lumbar lordosis (54%). The mean total FMS^TM^ score was 14.8. Most of the hockey players (57%) achieved a total FMS^TM^ score in the range of 14–17 points, whereas 28% had a total FMS^TM^ score of <14. Seventy-two percent of the studied athletes had at least one asymmetry. Significant differences between performing the movements on the right and the left sides of the body were observed in in-line lunges (p = 0.019) and shoulder mobility sub-tests (p < 0.001). The FMS^TM^ sub-tests performed with the lowest success rates were rotatory stability and the hurdle step. A lower score in the rotatory stability test is related to shoulder pain. It is highly important to develop appropriate exercise programs to reduce or prevent muscle imbalances in ice hockey players.

## Introduction

Competitive sports training requires the engagement of specific muscle groups with varying intensity, in specific positions, for an extended period of time and, if movements are asymmetrical, excessive, one-sided activity of muscles occurs. The exposure to intensive athletic training may affect spinal alignment, lead to a greater prevalence of morphological and functional asymmetries of the body, cause an overload in the osteoarticular and muscular system and the occurrence of specific movement patterns, and lead to functional deficits (Grabara, 2014, 2017; Krzykała et al., 2018; López-Miñarro et al., 2011). Muscular imbalances and overloads can induce compensations, which lead to an increased risk of injury, weakening players’ motor abilities and their mental state, affecting their availability, and resulting in the fear of further injuries (Kiesel et al., 2009; Mucha et al., 2016). Deviation is a change in the normal balancing effect of muscle synergists. Posture assessment screening can demonstrate the occurrence of muscles disorders that may be associated with movement impairments (Watson, 2001).

Ice hockey is a fast and dynamic sports discipline with frequent changes of directions and collisions, which are determined by trunk stability and strength (Kokinda et al., 2018; Upjohn et al., 2008). The high strain on the motor system of ice hockey players is reflected in musculoskeletal disorders, pain of the motor apparatus, asymmetries and muscle imbalances, as well as a high risk of injury (Brunner et al., 2020; Nordstrøm et al., 2020).

Ice hockey is categorized as a unilateral sport because of the preferential use of one side of the body (Bussey, 2010). Ice hockey players need to hold the stick on their dominant side and adopt a semiflexed position combined with the side bending and rotation, which must be sustained for most of the game. Such lateral dominant sports have been associated with an increased presence of functional asymmetries (right to left asymmetry) as compared with bilateral sports (Bussey, 2010). The side-specific position may affect tightens of the lower limbs and the back, which may lead to dysfunction and increase the risk of injury. Musculoskeletal injuries in professional ice hockey players are very common, with a lifetime incidence of lower back pain (LBP) ranging from 54% to 88% (Baranto et al., 2009; Fett et al., 2017) and 32–41% of players reporting a lower limb injury (LLI) during the competitive season. It has been found that increased asymmetry negatively affects physical performance (Resta et al., 2022).

The Functional Movement Screen (FMS^TM^) is a screening test of seven movement patterns, it can indicate which movement patterns are performed incorrectly, assess body posture and ranges of motion (Kiesel et al., 2007, 2009). The FMS^TM^ identifies deficits in seven movement tasks containing global, mobility and stability patterns (Cook et al., 2014; Rowan et al., 2015). In team sports, FMS^TM^ is a common way to evaluate the functional level of the team and determine training programs (Kiesel et al., 2009). FMS^TM^ may also provide a tool to assist in determining readiness to return to training and the completion of rehabilitation after injury or surgery (Cook et al., 2014).

Previous studies mainly focused on testing functional movement patterns in youth athletes and recreational athletes or on examining the association between the FMS^TM^ score and injuries (Chalmers et al., 2017; Kraus et al., 2014; Parenteau-Goudreault et al., 2014). To the best of our knowledge, there have been a small number of studies examining movement patterns or spinal posture in ice hockey players (Rowan et al., 2015).

Therefore, the aim of this study was to evaluate functional movement patterns and spinal posture of elite ice hockey players and to examine the association between spinal posture, the prevalence of musculoskeletal symptoms and FMS^TM^ scores.

We hypothesized that the FMS^TM^ test may indicate asymmetries among ice-hockey players. Abnormal and asymmetric posture can affect movement patterns. Therefore, we assumed that for most hockey players the FMS^TM^ score may be close to the cutoff point of 14. Due to the flexion position of the trunk and hips, and shoulder protraction, it can be assumed that the thoracic kyphosis may be increased, and the lumbar lordosis may be decreased.

## Methods

### Design and Procedures

A cross-sectional study design was used to investigate functional movement patterns, spinal posture, and the prevalence of musculoskeletal symptoms. The study was conducted in sports clubs before the off-ice warm up and ice practice.

### Participants

The study included 86 elite, professional male ice hockey players aged 18 to 38 years (mean 24.5 ± 6.1). The training experience of the examined athletes was 9 to 30 years (mean 16.6 ± 6.2). Almost half of study participants were national team players. The inclusion criteria were: consent to participate in the study, active participation in all training sessions. Participants were excluded from the study if they had acute musculoskeletal injuries that could interfere with their ability to participate in regular training, ill-being on the day of the examination, and players subjected to rehabilitation. The hockey players had not been tested by FMS^TM^ before and were not familiar with FMS^TM^ tests.

The study was approved by the Bioethics Committee of the Jerzy Kukuczka Academy of Physical Education in Katowice (certificate of approval No. KB 2/2017) and conformed to the standards set by the Declaration of Helsinki. All participants were informed about the type and the aim of the study, and they provided written informed consent before participating in the study.

### Measures

The study participants underwent anthropometric measurements, spinal posture assessment, FMS^TM^ testing, and they filled out a questionnaire.

Anthropometric assessments included body height (BH) measurements and body mass (BM). BM and fat mass [%] were assessed with electrical impedance (Tanita BC-418 body composition analyzer). The body mass index (BMI) was calculated based on BH and BM measurements.

Sagittal spinal curvatures were measured with a Saunders Baseline Digital Inclinometer (The Saunders Group Inc, Chaska, MN, USA). Each participant was examined while standing in a habitual posture. Their lower limbs were extended at the knee joint, with the feet hip-width apart, barefoot. The angle of thoracic kyphosis was measured by resetting the inclinometer at the Th_12_–L_1_ point and setting it to the C_7_–Th_1_ point. The angle of lumbar lordosis was measured by resetting the inclinometer at the L_5_–S_1_ point and setting it at Th_12_–L_1_ point. Each measurement was taken thrice, and the average value was used for analysis (Czaprowski et al., 2017). A value of 30–40 was accepted as a normal range of both thoracic kyphosis and lumbar lordosis (Walicka-Cupryś et al., 2019).

Functional movements patterns were assessed by the FMS^TM^. The FMS^TM^ was used to rank and grade movement patterns and to assist in the identification of functional limitations and asymmetries. As outlined by Cook et al. (2006) the FMS^TM^ included seven sub-tests for assessing range of motion, stability and symmetry presented in the order of execution: a deep squat, a hurdle step, an in-line lunge, shoulder mobility, an active straight leg raise, a trunk stability push up, and rotary stability (Cook et al., 2006). Five of them (i.e., number 2, 3, 4, 5 and 7) were performed bilaterally and detected possible asymmetries. In addition to the seven basic tests, the FMS^TM^ includes three provocation tests that are performed before the three tests (i.e., 4, 6 and 7). Participants received a score of zero to three if: they felt pain during the tested movement (0); did not perform the movement correctly (1); performed the tested movement with compensations (2); performed the correct tested movement (3). The score of all sub-tests was summed to provide a maximum score of 21 points. Asymmetric tests were assessed separately for the right and left sides, and if the results differed, the lower score was taken as the final result of that test. Participants had 3 attempts at each sub-test and the best score was registered (Cook et al., 2014). The overall assessment of the FMS^TM^ was carried out by a physiotherapist with a certificate of completing the course and passing the FMS exam, and with 7 years of experience. A previous study showed that the FMS^TM^ is a reliable test for youth elite hockey players, and the authors calculated an intra-class correlation coefficient (ICC) for the total score, with an ICC of 0.96 (Parenteau-Goudreault et al., 2014).

Musculoskeletal problems were assessed by the Nordic Musculoskeletal Questionnaire (NMQ). The NMQ quantifies musculoskeletal pain and activity prevention in nine body regions: the neck, shoulders, the upper back, elbows, the low back, wrists/hands, hips/thighs, knees and ankles/feet. The NMQ included a body map to indicate areas of the body causing musculoskeletal pain. Participants provided information on the presence or absence of pain in the past 12 months and in the past seven days separately for each of the nine body areas. The NMQ was also augmented with a numerical pain intensity rating scale that allowed participants to estimate pain intensity from 1 to 10 (from minimal pain to intense or unbearable pain). If participants reported pain in specific body areas in the past seven days, they also indicated the intensity of that pain on a scale from 1 to 10 (Grabara and Sadowska-Krępa, 2021; Kuorinka et al., 1987). The NMQ was also applied in the athletic population, and had appropriate validity and reliability with an ICC > 0.7 (Khorzoghi et al., 2021).

The research was carried out in December during the Polish hockey league's season. All hockey players were tested once, during the same period.

### Statistical Analysis

The statistical analysis was performed by Statistica v. 10 software (Statsoft Inc., USA). The results are expressed as means, standard deviations (mean ± SD), and median. The scores of asymmetric trials of the FMS^TM^ were analyzed by the Wilcoxon signed-rank test. The differences in FMS^TM^ scores according to the occurrence of musculoskeletal pain for nine anatomical regions in the last 7 days or the last 12 months were analyzed using the Mann-Whitney U test. A correlation analysis for angles of sagittal spinal curvatures and the FMS^TM^ scores was carried out by the Spearman correlation coefficient. The level of significance was set at 0.05 for all analyses.

## Results

The descriptive analysis for anthropometric variables and spinal curvatures is presented in Table 1. With respect to the accepted norms for the angles of thoracic kyphosis and lumbar lordosis, half of the studied ice hockey players had flattened lumbar lordosis. The thoracic kyphosis was within accepted norms (46%) or was hyperkyphosis (41%) (Table 1).

**Table 1:** Anthropometric variables and angles of sagittal spinal curvatures in ice hockey players.

Variables	Mean ± SD	Median	Percentage of players per norm category* (n = 86)		
			Below the norm	Norm	Above the norm
Body height [cm]	182.9 ± 4.72	183			
Body mass [kg]	83.7 ± 6.92	84			
Fat [%]	14.1 ± 3.45	14.6			
BMI [kg/m^2^]	25.63 ± 1.91	25.69			
Thoracic kyphosis angle [^0^]	38.3 ± 7.51	39.8	13%	46%	41%
Lumbar lordosis angle [^0^]	29.8 ± 6.85	28.3	54%	44%	2%

*Normal range for thoracic kyphosis and lumbar lordosis is 30^0^–40^0^ *(Walicka-Cupryś et al., 2019)*.

The descriptive analysis for FMS^TM^ sub-tests and total scores is presented in Table 2. A total FMS^TM^ score was relatively low. Of all tested ice hockey players, 24 (28%) of them had a total FMS^TM^ score of < 14. 49 (57%), almost of participants had a total score of 14–17, and only 13 (15%) of participants had a total score of >17.

Regarding the asymmetric FMS^TM^ trials, 24 (28%) of participants had no asymmetries, 42 (49%) had one asymmetry, 14 (16%) had two asymmetries, and 6 (7%) had three asymmetries. The analysis revealed statistically significant differences between right and left body sides in in-line lunges (*p* = 0.019) and shoulder mobility (*p* < 0.001) sub-tests. In the in-line lunge, a higher score was achieved for the right leg than the left leg, and in shoulder mobility, the better score was achieved when the right hand was elevated.

**Table 2:** The descriptive analysis for FMS^TM^ sub-tests and total scores [points] in ice hockey players.

FMS^TM^ sub-tests	Number and percentage of players per score category (n = 86)				Mean ± SD	Median
	0	1	2	3		
Deep squat	0 (0%)	11 (13%)	61 (71%)	14 (16%)	2.03 ± 0.54	2
Hurdle step	0 (0%)	19 (22%)	54 (63%)	13 (15%)	1.93 ± 0.61	2
In-line lunge	0 (0%)	13 (15%)	53 (62%)	20 (23%)	2.08 ± 0.62	2
Active straight leg rise	0 (0%)	3 (4%)	52 (60%)	31 (36%)	2.33 ± 0.54	2
Shoulder mobility	1 (1%)	12 (14%)	33 (38%)	40 (47%)	2.3 ± 0.75	2
Trunk stability push-up	1 (1%)	3 (3.5%)	46 (53.5%)	36 (42%)	2.36 ± 0.61	2
Rotary stability	1 (1%)	19 (22%)	65 (76%)	1 (1%)	1.77 ± 0.48	2
FMS^TM^ total score					14.8 ± 2.38	15

*Note: 0 – pain during the tested movement; 1 – lack of the correct tested movement; 2 – the tested movement with compensation; 3 – the correct tested movement*

Eighty % of players reported lower back pain and 47% of all participants reported knee pain over the previous 12 months (Table 3).

**Table 3:** Observed prevalence rates for neck/back pain and other musculoskeletal pain in elite hockey players (n = 86).

Area of the body affected	Occurrence in past 12 months n (%)	Occurrence in past 7 days n (%)	Pain intensity for past 7 days on a scale of 1–10 (min–max)	Pain intensity for past 7 days on a scale of 1–10 (mean ± SD)
Neck	22 (25.6%)	14 (16.3%)	1–9	4 ± 2.18
Shoulder(s)	24 (27.9%)	13 (15.1%)	3–7	4.7 ± 1.3
Upper back	29 (33.7%)	17 (19.8%)	1–6	3.56 ± 1.29
Elbow(s)	13 (15.1%)	4 (4.7%)	3–7	5.25 ± 2.06
Wrist(s)/hand(s)	29 (33.7%)	19 (22.1%)	2–8	3.8 ± 1.7
Lower back	69 (80.2%)	67 (77.9%)	2–8	4.4 ± 1.35
Hip(s)/thigh(s)	14 (16.3%)	9 (10.5%)	1–8	4 ± 2.12
Knee(s)	41 (47.7%)	25 (29.1%)	1–8	4.2 ± 1.85
Ankle(s)/feet	19 (22.1%)	13 (15.1%)	2–10	4.54 ± 2.3

The differences in FMS^TM^ scores between players who reported and who did not report musculoskeletal pain are shown in Table 4. The results indicate that shoulder mobility was lower in players reporting upper back pain and/or shoulder problems during the last 7 days compared to players who did not report these symptoms. Athletes who complained of neck and/or shoulder pain during the last seven days received less points in the rotatory stability test than athletes who did not report these problems. Athletes who reported upper back pain during the last 12 months received less points in the active straight leg rise test compared to players who did not report upper back pain.

**Table 4:** The differences in FMS^TM^ scores between elite hockey players who reported and who did not report the musculoskeletal pain.

FMS^TM^ sub-tests	Area of the body affected					
	Upper back pain 12	Low back pain 12	Neck pain 7	Shoulders pain 7	Upper back pain 7	Low back pain 7
Hurdle step L	Z = 2.69 *p* = 0.007		Z = 1.97 *p* = 0.049			
In-line lunge L		Z = 2.29 *p* = 0.022				Z = 2.13 *p* = 0.033
Shoulder mobility L	Z = 1.97 *p* = 0.048			Z = 2.24 *p* = 0.025	Z = 2.16 *p* = 0.031	
Shoulder mobility F	Z = 1.97 *p* = 0.048			Z = 2.24 *p* = 0.025	Z = 2.16 *p* = 0.031	
Active straight leg raise L	Z = 2.39 *p* = 0.017					
Active straight leg raise R	Z = 2.39 *p* = 0.017					
Active straight leg raise F	Z = 2.26 *p* = 0.024					
Rotatory stability L			Z = 2.18 *p* = 0.029	Z = 2.38 *p* = 0.017		
Rotatory stability R			Z = 2.03 *p* = 0.042			
Rotatory stability F				Z = 2.07 *p* = 0.038		
Final score of FMS^TM^	Z = 2.38 *p* = 0.017					

*Note: Only statistically significant differences are shown; L – left side; R – right side; F – final score; 12 – occurrence of pain during past 12 months; 7 – occurrence of pain during past 7 days*

The correlation analysis of spinal posture and FMS^TM^ scores showed a relationship between thoracic kyphosis angle and shoulder mobility (r = −0.22; *p* = 0.042). It was observed that, with the increase in the kyphosis angle, shoulder mobility decreased.

## Discussion

We investigated functional movement patterns and spinal posture of elite ice hockey players and associations between spinal posture, the prevalence of musculoskeletal symptoms and FMS^TM^ scores.

Our study revealed a relatively low total FMS^TM^ score. The FMS^TM^ test is a screening test that is generic to all disciplines. A low score among ice hockey players may be due to a specific asymmetrical position or muscle imbalance, which can cause the tested movements to be performed with compensation. According to Kiesel et al. (2007), the lower composite score (<14) on the FMS^TM^ increases the risk of injury. Therefore, 28% of our participants can be considered at risk for an injury. However, Dorrel et al. (2015, 2018) observed a non-significant relationship between FMS^TM^ scores of ≤15 and injuries. Those authors indicated that the FMS^TM^ provided low sensitivity for discrimination of high injury risk and stated that FMS^TM^ had limited prognostic ability to identify the risk of injury. Regardless of that, the FMS^TM^ is still an appropriate tool to identify deficits that may increase the risk of injuries or indicate inadequate recovery from a prior injury (Dorrel et al., 2015, 2018).

As we assumed, the mean of the total FMS^TM^ score of studied hockey players indicates a low level of the tested movement patterns performance. The results are consistent with those reported by Dossa et al.’s (2014) study who noted 14.7 as the FMS^TM^ mean total score in major junior hockey players, and by Rowan et al. (2015) who examined 81 elite junior (17–19 years old) hockey players, and noted 15.2 as the FMS^TM^ mean total score. A slightly higher mean total result of the FMS^TM^ (15.76) was found by Boguszewski et al. (2017) in male youth hockey players, and this result was significantly higher in hockey players with a training experience of nearly five years than in non-athlete participants.

Rotatory stability and the hurdle step were the movements performed with the lowest success rates by the studied athletes. Only one hockey player got three points for the rotatory stability test. This pattern is complex, requiring proper neuromuscular coordination and energy transfer through the torso. The test represents the coordinated efforts of mobility and stability. Pelvis, core, and shoulder girdle stability can be evaluated during this test (Cook et al., 2014). Improper performance of this test may be connected with the problems of the lumbar-pelvic-femoral complex. A large percentage of lower back problems occurs because abdominal muscles do not maintain control over the rotation between the pelvis and the spine at the L_5_-S_1_ level. The rotational stability test requires a stable position and correct muscle timing on both sides when performing rotational movements. The ice hockey position is set asymmetrically due to the specific stick holding, which can already affect the deep muscle imbalance between the right and left sides, including the multifidus muscle which has the function of stabilization and proprioception of the lumbar spine (Fortin et al., 2019). Core muscles are actively involved in almost every aspect of ice hockey. Due to the asymmetrical position, an imbalance can form, leading to eccentric contraction on one side (Lee et al., 2006).

The hurdle step test requires proper coordination and stability between the hips and the torso during the stepping motion, as well as single leg stance ability. The hurdle step also assesses bilateral functional mobility and stability of the hips, knees, and ankles. The test requires maximum flexion of the hip of one leg while maintaining the stability of the upright second hip, which requires good hip mobility and the cooperation of all hip controlling muscles (Cook et al., 2014). A test performed below three points may indicate problems with mobility and stability. Previous studies have indicated that hip mobility problems are a common weakness of hockey players (Wilcox et al., 2015; Wörner et al., 2019). Hip flexion may be limited by the asymmetrical flexion position during skating (Kuhn et al., 2016). Optimal hip flexion of more than 90° is required to score three points in the hurdle step test, while the tested athletes often compensate this movement by positioning the hip in external rotation while going over the hurdle. Ice hockey players had the highest prevalence of femoroacetabular impingement (FAI) which significantly reduces the rotational movements of the hip joints, especially flexion and internal rotation, and may disrupt the biomechanics of the lower limbs and the trunk (Brunner et al., 2016; Doran et al., 2022).

The other low FMS^TM^ sub-test scores were registered in the deep squat and in the in-line-lunge. The deep squat pattern is used to examine mobility, postural control, pelvic and core stability. If performed correctly, the deep squat demonstrates fully coordinated extremity mobility and core stability, while the hips and shoulders function in symmetrical positions (Cook et al., 2014). The significance of the correct implementation of the squat pattern has been shown in other studies (Kritz et al., 2009; Wisløff et al., 2004). Lower extremity movements is the area that needs improvement in hockey players, especially the hip was found to be a probable area to be injured (Parenteau-Goudreault et al., 2014; Rowan et al., 2015). While sprinting, ice hockey players externally rotate in abduction during the push-off phase and internally rotate through increasing hip flexion during the recovery phase, displaying the at-risk hip positions of the ice hockey skating stride (Stull et al., 2011). Deep hip flexion during squatting, as well as skating movements on ice, can cause hip impingement (Stull et al., 2011). Ice hockey players are therefore at high risk of developing FAI (Brunner et al., 2016). Reduced hip range of motion due to FAI can lead to a low FMS^TM^ score in the deep squat as well as in the in-line lunge.

The in-line lunge test assesses hip and ankle mobility and stability, quadriceps flexibility, and knee stability. The in-line lunge pattern tests require balancing in a narrow position, and continued dynamic control of the pelvis and core within an asymmetrical hip position equally sharing the load (Cook et al., 2014). The present study revealed significant differences in performing the in-line lunge test on the right and left sides. Because of the imbalances, the hip joint does not move properly and compensations may appear in basic movements (Boyle et al., 2016).

Low mobility of the ankle and hip joints can overload the knee joints. Nearly half of the studied hockey players also reported knee pain. Lack of rotational hip mobility affects the knee joint and may increase the risk of non-contact anterior cruciate ligament (ACL) injury (VandenBerg et al., 2017). A previous study reported also that ice hockey players with a reduction in hip abduction range of motion have a greater prevalence of LBP (Fett et al., 2017).

The FMS^TM^ sub-tests performed with the highest success rates were as follows: a trunk stability push-up, an active straight leg rise, and shoulder mobility. The study including elite junior ice hockey players revealed that the best results of the FMS^TM^ sub-test were in the trunk stability push-up test, the in-line lunge test, and the active leg raise test (Rowan et al., 2015). These results were similar to our observations.

We also found significant differences in shoulder mobility between the right and left sides, and we noticed that shoulder mobility was worse in players reporting upper back pain and/or shoulder problems during the last seven days than in players who did not report these problems. Fifty % of professional field hockey players had hypertonic upper body muscles with a right-hand side bias, which could explain our findings (Kawałek and Garsztka, 2013). The same asymmetry was also found in male ice hockey players at a mean age of 12.5 years (Boguszewski et al., 2017).

It was hypothesized that the specific position of a hockey player with trunk and hip flexion and shoulder protraction adopting while skating can affect the posture. As we assumed, spinal posture of studied ice hockey players was characterized by decreased lumbar lordosis, whereas thoracic kyphosis was often in the normal range or was increased. The abnormal lumbar lordosis may contribute to low back pain (Mirbagheri et al., 2015). We noticed that most of players reported LBP. We also observed that when the kyphosis angle increased, shoulder mobility decreased.

To the best of our knowledge, there have been no other studies involving assessment of antero-posterior curvatures in adult male ice hockey players. Therefore, it is difficult to determine if ice hockey may predispose players to variability of spinal curvatures in the sagittal plane. Rajabi et al. (2012) found a significantly increased rate of thoracic kyphosis in adolescent female field hockey players compared to non-athlete controls. Flis-Masłowska et al. (2018) also observed an increase in the physiological lumbar lordosis in field hockey players when compared to the control group.

Previous studies concerning athlete’s posture have shown that sports training affects spinal curvatures and alignment of the shoulder girdle, scapulae, and pelvis due to specific movement patterns. A high level of athletic training could lead to a decrease in the lateral deviation of the spine (Betsch et al., 2015), and in ice hockey players, to a tendency for left-sided scoliosis (Mucha et al., 2016). Previous studies have observed scapular posture asymmetry between the dominant and non-dominant sides and the prevalence of asymmetries of the shoulders and the pelvis in athletes representing overhead sports, such as volleyball and tennis (Barczyk-Pawelec et al., 2012; Oyama et al., 2008; Ribeiro and Pascoal, 2013). The study on a large group of male athletes (rugby, soccer, kendo, swimming, bodybuilding, several track and field events: sprinting, distance running, jumping and throwing) showed that spinal curvatures were sport-specific (Uetake et al., 1998).

## Strength and Limitations of the Study

The study provides new information on an important sports training topic: the relationship between functional movement patterns, spinal posture, and the prevalence of musculoskeletal symptoms of elite ice hockey players. Our research included hockey players from three teams of the Polish Premier League in ice hockey. This work fills a research niche in the topic.

There are several limitations of this study. First, its cross-sectional nature, second, spinal curvatures were only measured while standing in a habitual posture. Third, the FMS^TM^ is a screening test which does not allow for diagnosis, but only indicates certain limitations and asymmetries. The other limitation of this study is the lack of a control group to which the results of the FMS^TM^ and the anteroposterior spinal curvatures might be compared. Another limitation is that the NMQ was handed out once, and since it refers to the past seven days and the past year, it can be difficult for participants to capture the frequency, intensity, or location of pain. The final limitation is the lack of data related to previous injuries of players.

## Conclusions

Our results revealed a relatively low (14.8) total FMS^TM^ score in the studied elite ice hockey players. The FMS^TM^ sub-tests performed with the lowest success rates were rotatory stability and the hurdle step. The lower score in the rotatory stability test was connected with the occurrence of shoulder pain.

We also observed significant differences between performing the movements on the right and the left body side in the in-line lunge and shoulder mobility sub-tests.

Finally, the spinal posture of the studied ice hockey players was characterized by decreased lumbar lordosis, whereas thoracic kyphosis was often in the normal range or was increased, and the result in thoracic kyphosis was related to a decrease in shoulder mobility.
